# Publisher Correction: Hydrophobic gating in BK channels

**DOI:** 10.1038/s41467-020-18517-2

**Published:** 2020-09-03

**Authors:** Zhiguang Jia, Mahdieh Yazdani, Guohui Zhang, Jianmin Cui, Jianhan Chen

**Affiliations:** 1 Department of Chemistry, University of Massachusetts, Amherst, MA 01003 USA; 2 Department of Biomedical Engineering, Center for the Investigation of Membrane Excitability Disorders, Cardiac Bioelectricity and Arrhythmia Center, Washington University, St Louis, MO 63130 USA; 3 Department of Biochemistry and Molecular Biology, University of Massachusetts, Amherst, MA 01003 USA

**Keywords:** Computational biophysics, Permeation and transport

Correction to: *Nature Communications* 10.1038/s41467-018-05970-3, published online 24 August 2018.

The original version of this article contained an error in the Methods section, which incorrectly read ‘nuclear nonproliferation treaty’. The correct version removes this phrase. This has been corrected in both the PDF and HTML versions of the article.

