# Supplementary material for: Hydrophobic gating in BK channels
Source: Nat Commun. 2018 Aug 24;9:3408. doi: 10.1038/s41467-018-05970-3 (PMC6109084; doi:10.1038/s41467-018-05970-3)
Supplement: Supplementary file 1 — Supplementary Information [file 41467_2018_5970_MOESM1_ESM.pdf]

Supplementary Information

Hydrophobic Gating in BK Channels

Zhiguang Jia[1], Mahdieh Yazdani[1], Guohui Zhang[3], Jianmin Cui[3], and Jianhan Chen[1,2*]

[1]Department of Chemistry and [2]Department of Biochemistry and Molecular Biology, University of Massachusetts, Amherst, MA 01003, USA

[3]Department of Biomedical Engineering, Center for the Investigation of Membrane Excitability Disorders, Cardiac Bioelectricity and Arrhythmia Center, Washington University, St Louis, MO 63130, USA

*Corresponding Author: Phone: 413-545-3386; Email: jianhanc@umass.edu

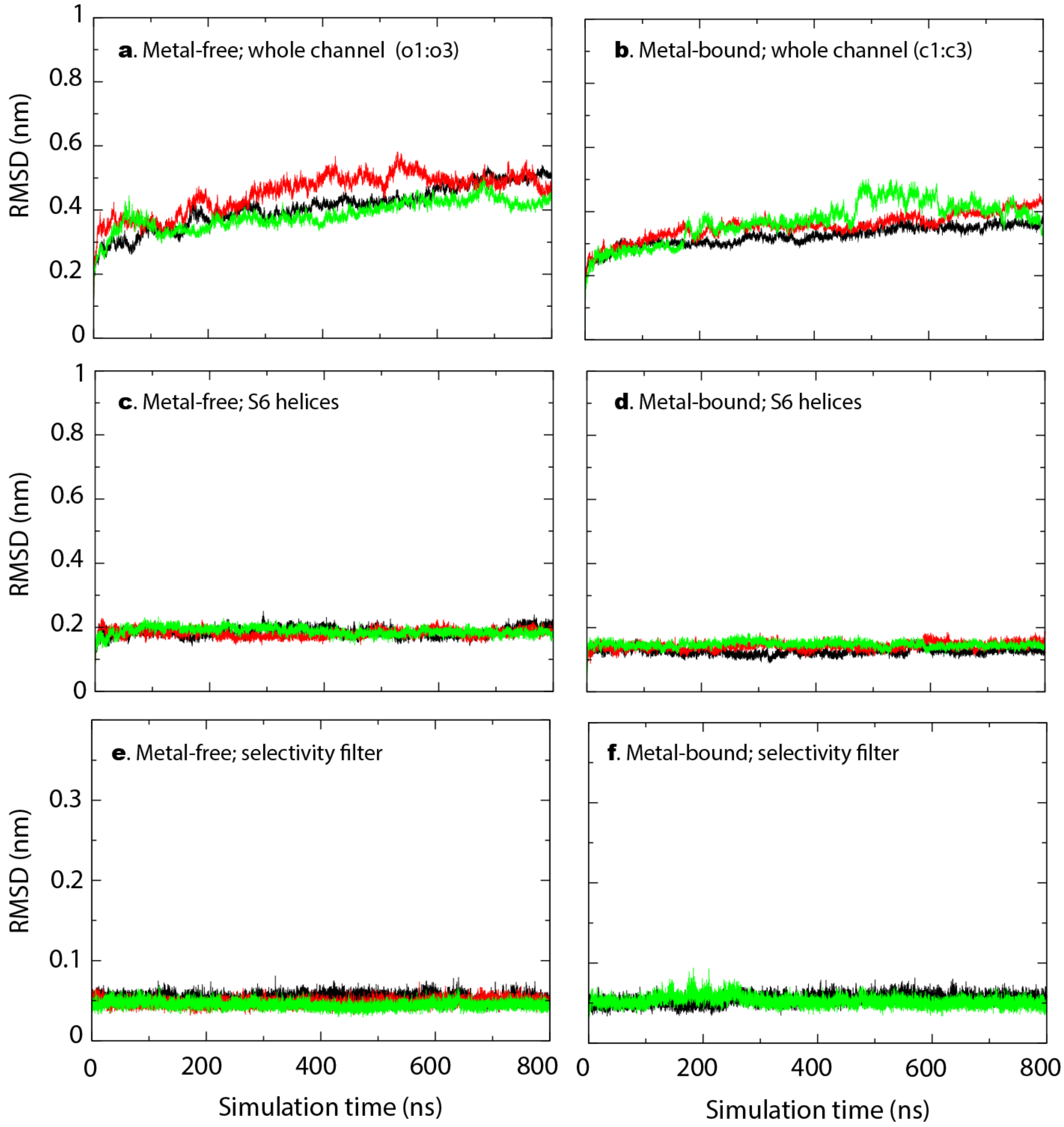

Supplementary Figure 1 Evolution of backbone RMSD values from the initial structures. Representative simulations are shown for human BK channels in metal-bound (*o1*, *o2* and *o3*) and metal-free (*c1*, *c2* and c3) states for the whole channel (a & b), pore-lining S6 helices (c & d), and the selectivity filter (S286 to D292) (e & f).

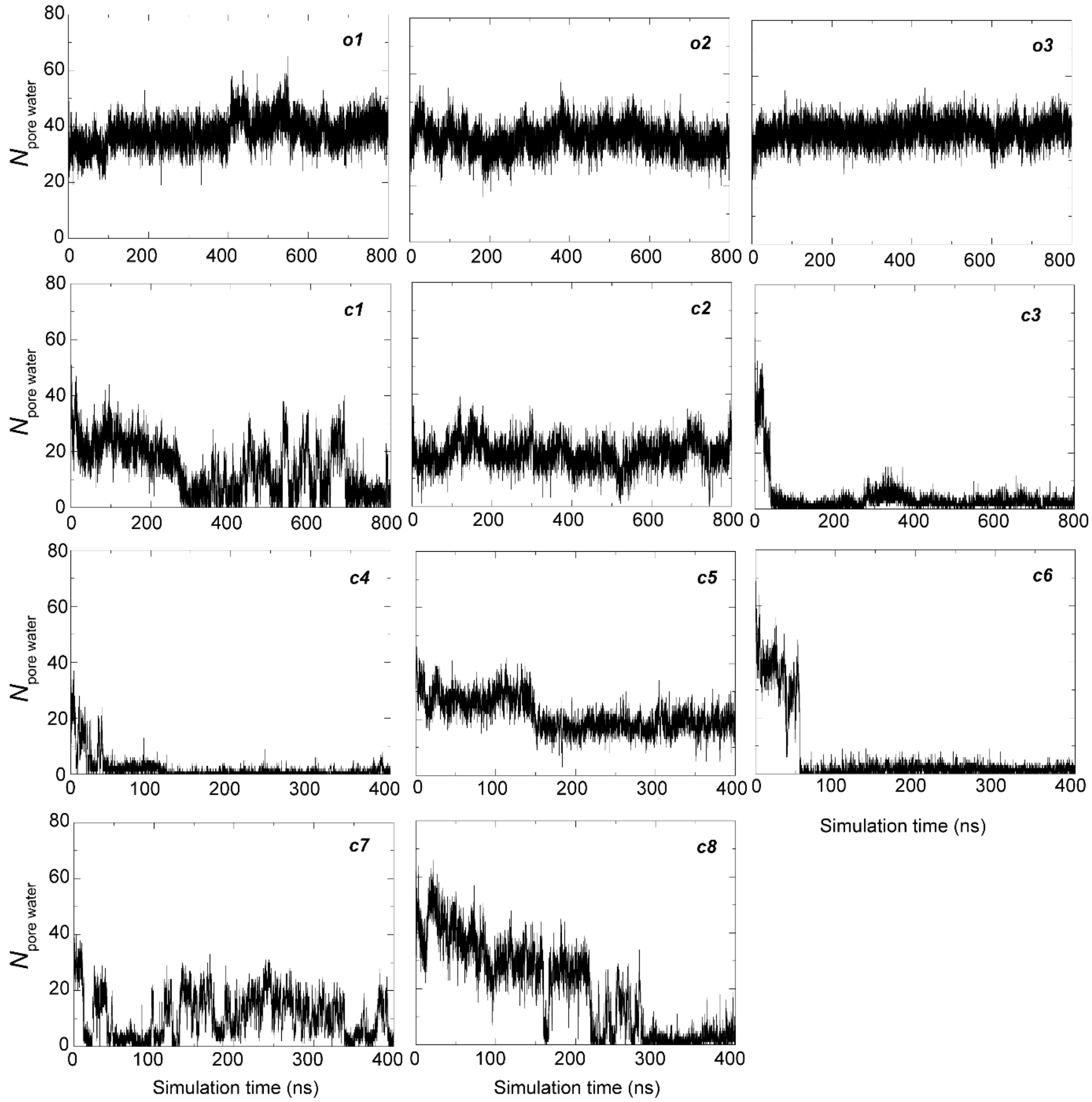

Supplementary Figure 2 Number of pore water molecules as a function of the simulation time. Results are shown for simulations of the metal-bound (*o1*-*o3*) and metal-free (*c1*-*c8*) human BK channels.

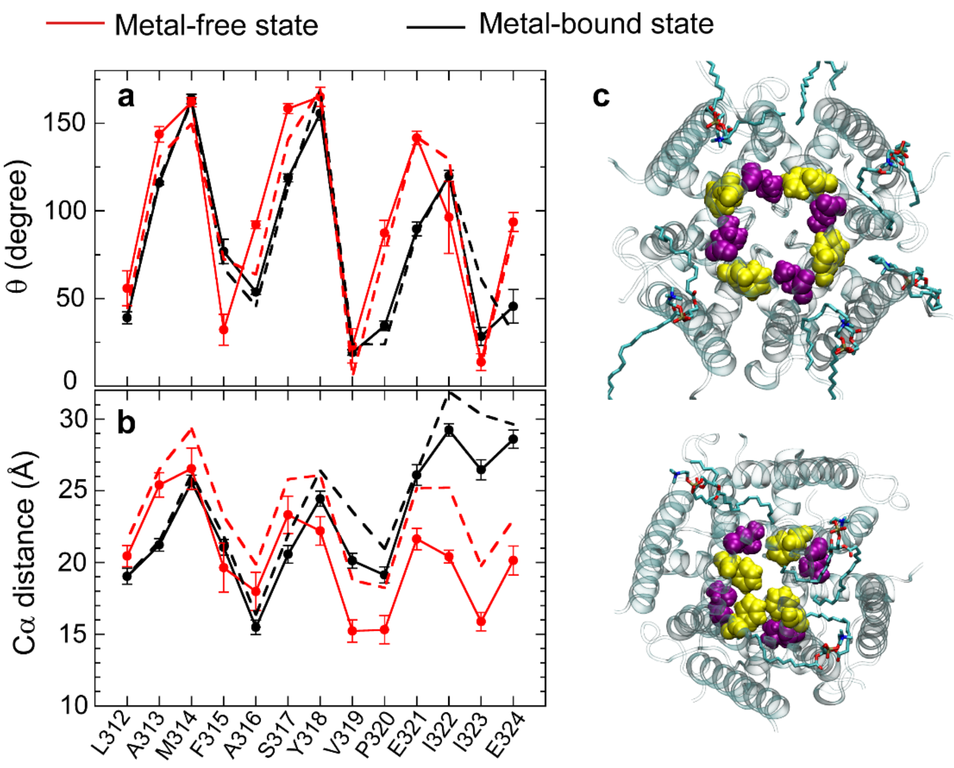

Supplementary Figure 3 Structural features of the S6 helix in the metal-bound and metal-free simulations. **a**, Orientation of residue sidechains along the S6 helix and **b**, Cα-Cα distance between the same residue on non-neighboring S6 helices in the MD equilibrated (solid traces) and homology modeled (dashed traces) structures of the metal-bound and metal-free channels. Smaller angles correspond to the cases of side chain pointing toward the center of the pore. **c,** Representative sidechain orientations of F315 (yellow spheres) and L312 (purple spheres) in the hydrated metal-bound (derived from simulation *o1* at 125 ns, top panel) and dewetted metal free (derived from simulation *c4*, at 88ns, bottom panel) states. Adjacent lipid molecules are shown as cyan sticks.

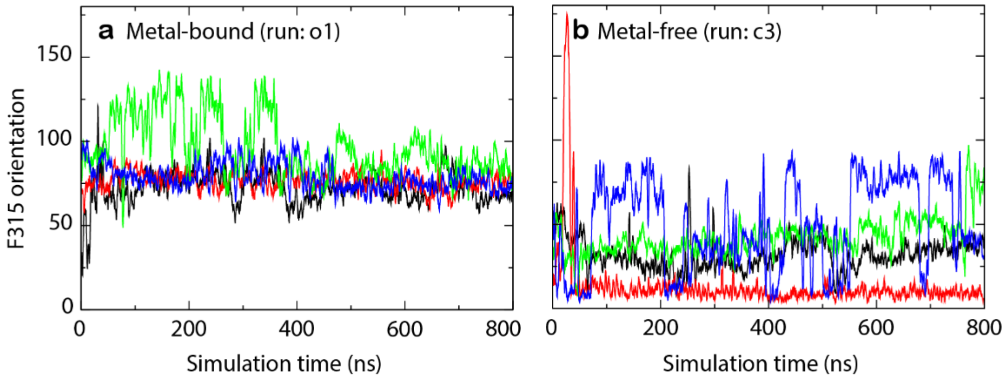

Supplementary Figure 4 Orientation F315 sidechains during simulations. Representative traces are shown for **a**, metal-bound and **b**, metal-free simulations. Each trace corresponds to one subunit of the tetramer. The orientation was calculated as the angle between two the Cα to pore center and Cα to sidechain center-of-mass vectors in in the x-y plane. Smaller angles correspond to pore pointing orientations. The results show that F315 orientation fluctuates substantially and independently in the dry state of the pore.

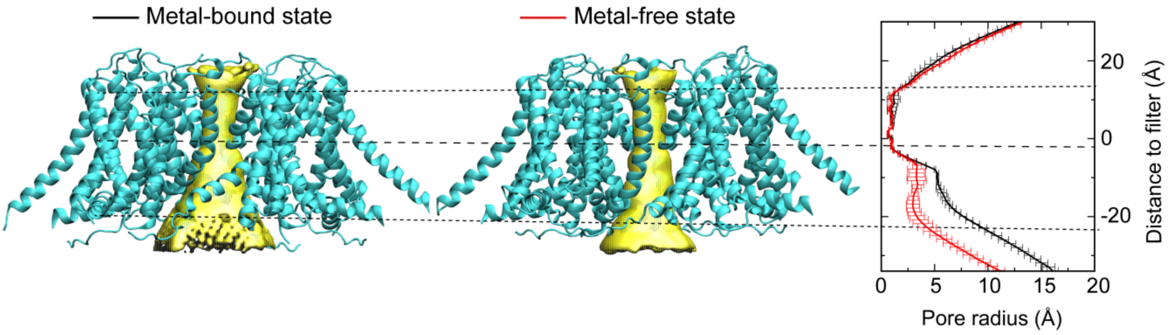

Supplementary Figure 5 Ion conduction pathways and pore profiles of TMD of the hBK channel derived from MD simulations. The results are averaged over 150-200 ns of simulation *c3*, *c4* and *c6* for the metal-free state and simulation *o1*, *o2* and *o3* for the metal-bound state. The standard deviations among the three runs are shown as the error bars.

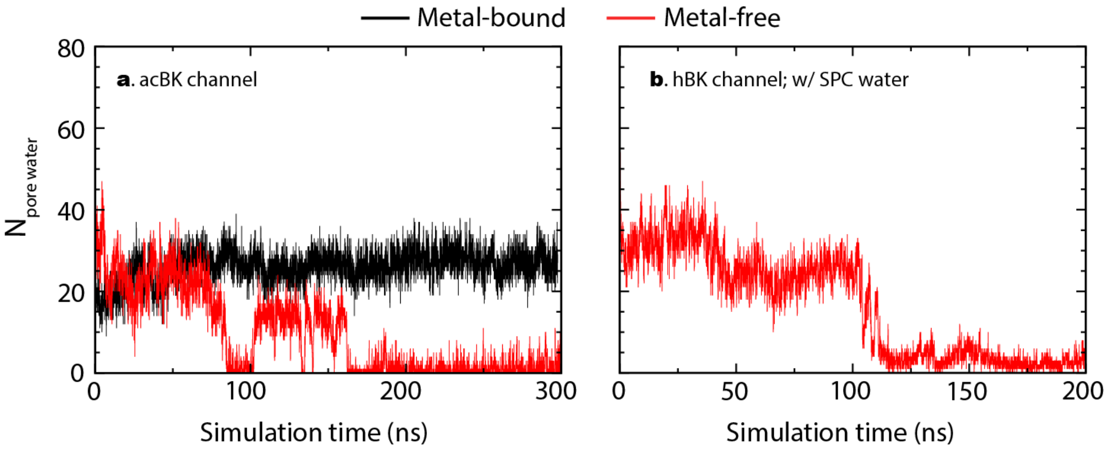

Supplementary Figure 6 Number of pore water molecules as a function of the simulation time. a) Metal-free (red trace) and metal-bound (black trace) *Aplysia* BK channel simulated using the CHARMM36m force field with TIP3P water; b) the metal-free hBK channel using the Gromos force field with SPC water (see the main text for details).

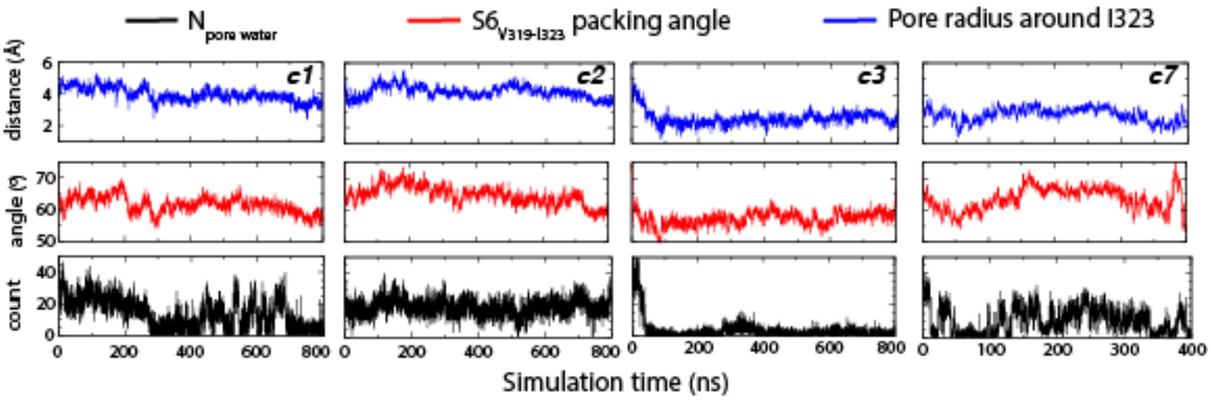

Supplementary Figure 7 Evolution of key pore structural properties during dewetting transitions. Four selected simulations (c1, c2, c3, c7) are shown. The S6 packing angle shown was calculated as the averaged cross angles between neighboring S6 V319 I323 segments, defined using the positions of Cα atoms of V319 and V323.

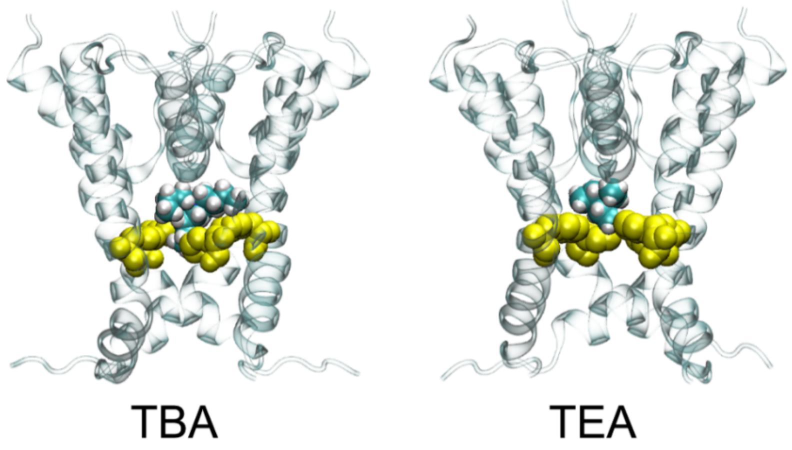

Supplementary Figure 8 Representative bound conformations of TBA and TEA in the deep pore region of the metal-bound hBK channel. TEA and TBA molecules are shown as van der Waals spheres and colored according to atom types. Shown as transparent cartoon is BK channel filter and S6 helices from three subunits with F315 shown as yellow van der Waals spheres. These snapshots are from umbrella sampling simulations and TEA/TBA is ~8 Å from the center of mass of the selectivity filter.

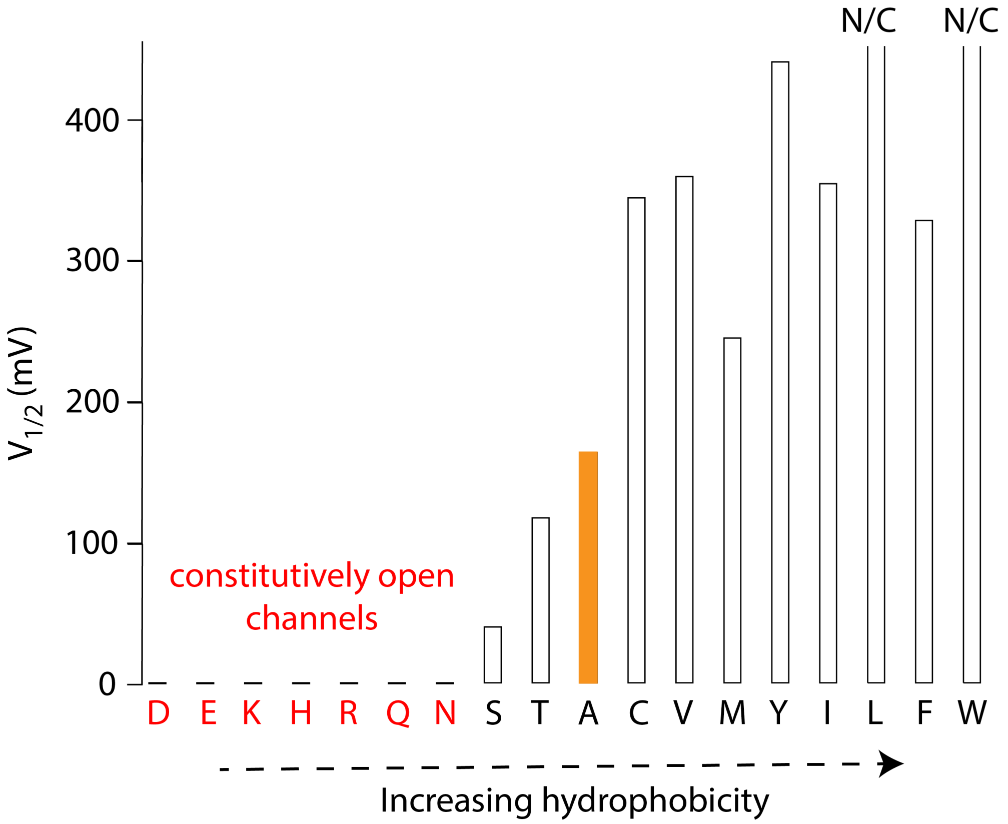

Supplementary Figure 9 Effects of single amino acid substitutions at position A316 on the half activation membrane voltage ($V_{1/2}$) of the hBK channel. The results were obtained from ref [1]. While charged/polar mutants favor the open state, hydrophobic mutants favor the close state and display increased $V_{1/2}$. For A316L and A316W mutants, the G-V curves are too right shifted or the currents are too small to reliably determine $V_{1/2}$ [1].

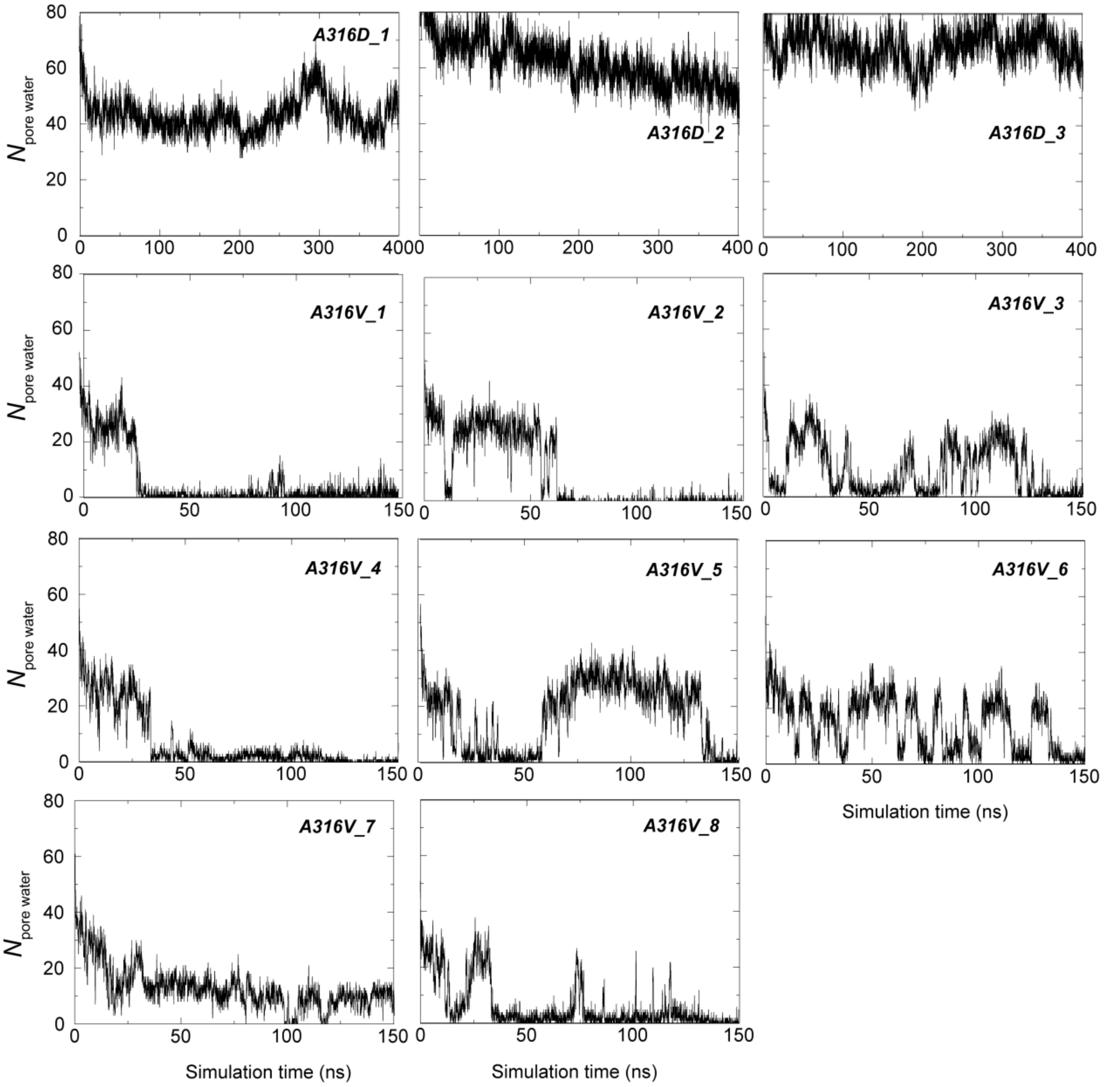

Supplementary Figure 10 Number of pore water molecules as a function of the simulation time for the metal-free hBK A316D or A316V mutant channels.

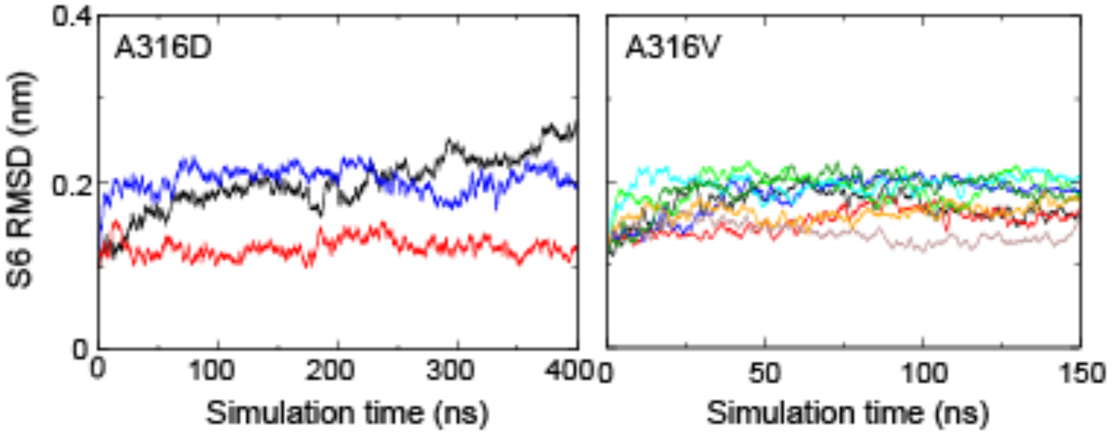

Supplementary Figure 11 Backbone RMSD of the pore lining S6 helices during simulations of A316D and A316V mutant BK channels in the metal-free state. Each traces represents a single run.

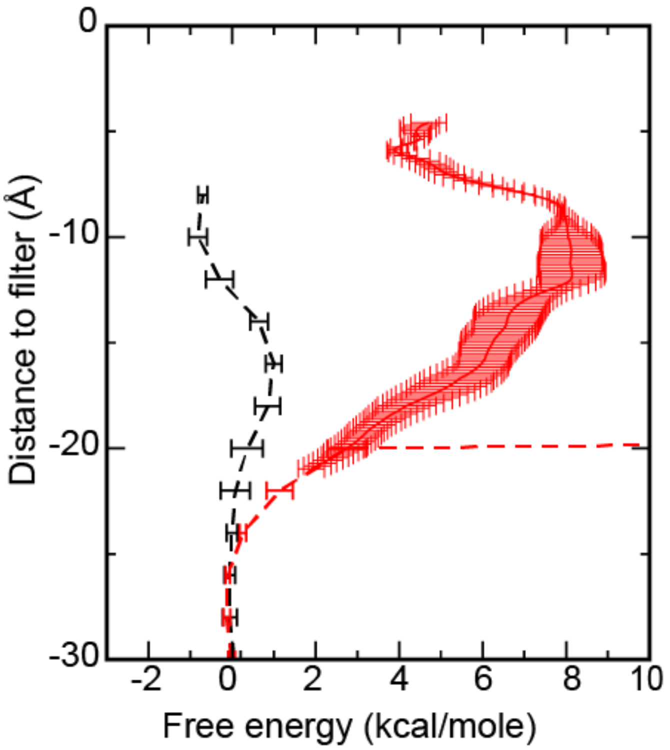

Supplementary Figure 12 Free-energy of pore permeation for K^+ in A316D (black traces) and A316V (red traces) in the metal-free state. The results calculated from umbrella sampling shown in solid traces, respectively. PMFs of K^+ derived from equilibrium simulations are shown in dashed lines. Error bars show standard errors between free-energy profiles from the first and second half of the data.

Supplementary References

1. Chen X, Yan J, Aldrich RW. BK channel opening involves side-chain reorientation of multiple deep-pore residues. *Proc. Natl. Acad. Sci. U.S.A.* **111**, E79-E88 (2014).
